# Comparative analysis of soil prokaryotic community structure and function in Ethiopian Church Forests and adjacent habitats

**DOI:** 10.1038/s41598-026-57528-9

**Published:** 2026-06-11

**Authors:** Biazen Endalamaw, Nigussie Haregeweyn, Takeshi Taniguchi, Getu Abebe, Masataka Nakayama, Mitsuru Tsubo, Menale Wondie, Takeshi Abe, Yoseph Buta Hailu, Atsushi Tsunekawa

**Affiliations:** 1 The United Graduate School of Agricultural Sciences, Tottori University, 4-101 Koyama- Minami, Tottori, 680-8553 Japan; 2 Forestry Research Department, Amhara Agricultural Research Institute, P.O. Box 527, Bahir Dar, Ethiopia; 3 International Platform for Dryland Research and Education, Tottori University, 1390 Hamasaka, 680-0001 Tottori, Japan; 4 Faculty of Environmental, Life, Natural Science and Technology, Okayama University, Okayama, Japan; 5 School of Civil and Water Resources Engineering, Debre Markos Institute of Technology, Debre Markos University, P.O. Box 269, Debre Markos, Ethiopia

**Keywords:** Dry afromontane forest, Functional potential, Habitat degradation, Indicator tree, Soil prokaryotes, Ecology, Ecology, Environmental sciences, Microbiology

## Abstract

Soil microorganisms are central to ecosystem functioning, yet how habitat degradation affects their community structure and functional potential in dry Afromontane forests (DAFs) remains poorly understood. This study investigated soil prokaryotic communities across five habitats representing a disturbance gradient in Ethiopian DAFs: Church forest, State forest, Community-managed forest, Shrubland, and Cropland. Using next-generation sequencing, we examined prokaryotic community composition, diversity, assembly processes, and predicted functional profiles. Actinobacteriota and Crenarchaeota were more abundant in less disturbed forests, whereas disturbed habitats showed shifts toward taxa associated with stressed conditions. Alpha diversity was lower in Church and State forests than in shrubland and cropland, indicating environmental filtering and dominance of habitat specialists. Community composition differed significantly among habitats and was associated with soil properties, stand attributes, and indicator trees. Deterministic processes explained most community turnover (81%), indicating strong environmental structuring, with homogeneous selection more evident in forests and mixed deterministic–stochastic processes in disturbed habitats. Predicted functional profiles suggest shifts in nitrogen-related processes, particularly ammonia oxidation and nitrification, although not directly measure microbial activity. Overall, habitat degradation is associated with changes in prokaryotic community structure and assembly, highlighting the importance of conserving intact forests and supporting restoration efforts in dryland ecosystems.

**Supplementary Information:**

The online version contains supplementary material available at https://doi.org/10.1038/s41598-026-57528-9.

## Introduction

Afromontane forests are among the most diverse ecosystem in the mountainous regions of sub-Saharan Africa, they are increasingly threatened by land-use change, climate stress, and expanding human activities^1,2^. Dry Afromontane forests (DAFs), in particular, have experienced extensive degradation and fragmentation, especially in northern Ethiopia, where agriculture and settlement have caused severe fragmentation^3,4^. In this landscape, Ethiopian Orthodox Tewahedo church forests (CFs) are among the last remnants of native DAF and have been preserved for centuries^5^. These forests maintain native biodiversity and key ecological processes such as nutrient cycling, and soil stabilization^6^, and serve as important reference system for ecosystem restoration.

Soil microbial communities are central to ecosystem functioning through their roles in organic matter decomposition, nutrient transformations, and plant–soil interactions^7^. Because microbial taxa differ in ecological strategies and environmental tolerances, changes in microbial community and diversity is often associated with variation in soil conditions, vegetation, and land-use history^8^. As soil microbes respond rapidly to environmental change, they are frequently used as indicators of ecosystem alteration across degradation and restoration gradients^9^. However, microbial diversity alone is not a consistent indicator of ecosystem conditions. Ecological theory suggests that less disturbed habitats may exhibit lower alpha diversity due to strong environmental filtering, whereas disturbed habitats may support higher diversity due to increased heterogeneity and the proliferation of generalist taxa^10,11^. In addition, microbial diversity and function are influenced by interacting environmental gradients and resource availability rather than disturbance alone^12,13^.

Despite their ecological importance, microbial responses to habitat degradation and restoration in DAF ecosystems remain poorly understood, with previous studies reporting inconsistent patterns of diversity change^14–20^. These inconsistencies likely reflect differences in environmental filtering, resource availability, and land-use history rather than uniform disturbance effects^10^. Habitat degradation alters forest structure (e.g., stand density, basal area, and species composition) and soil properties (e.g. nutrient content, cation balance, and structure), which are associated with shifts in microbial community composition and phylogenetic structure^21–24^. In addition, associations between microbial communities and indicator woody species may reflect shared responses to environmental gradients rather than direct causal relationships^25–27^. Soil nutrient availability can also affect microbial diversity and activity^24,28,29^, consistent with the “more individuals hypothesis”^30^. Accordingly, degradation- and restoration-driven changes in soil resources (e.g., C, N, Na⁺, Ca²⁺) restructure microbial assemblages primarily through shifts in habitat conditions^10,18,19,31^. These processes likely underline variation in soil microbial composition and function in transformed ecosystems such as DAFs.

Understanding the mechanisms underlying soil microbial assembly is crucial for predicting responses to environmental change^32^. Community assembly theory distinguishes between deterministic (e.g., environmental filtering and biotic interactions) and stochastic processes (e.g., dispersal limitation and ecological drift), both of which influence microbial composition^33–35^. Their relative contributions varies across the environmental contexts^35^ and remain poorly understood under long-term degradation or restoration. Phylogeny-informed null models offer a robust framework for disentangling these mechanisms^33,36–38^ and can clarify how disturbance and ecological succession are associated with shifts in microbial diversity and community structure. Researches in functional ecology and disturbance theory shows that environmental disturbances restructure biodiversity and functional traits^12,13,29,39^, underscoring the global importance of understanding how soil microbial communities influence ecosystem functioning and resilience. In parallel, taxonomic information derived from 16 S rRNA gene sequencing can be used to infer putative functional potential, providing complementary insight into potential ecosystem-level implications, although such inferences do not directly measure microbial activity^40^. Together, these approaches allow explicit testing of how environmental variables are associated with community composition, assembly mechanisms, and inferred functional shifts across disturbance gradients.

Here, we aimed to investigate how habitat degradation and restoration influence soil prokaryotic community composition, diversity, assembly processes, and taxonomically inferred functional potential in the DAFs of northern Ethiopia. The Tara Gedam CF landscape, a remnant reference habitats within the Ethiopian DAF^4^, provides an ideal setting for such an investigation. The CF serves as a genetic reservoir for indigenous tree species^5,6^, while the surrounding landscape has undergone experienced extensive degradation from deforestation, overgrazing, and agricultural expansion^41^. To counter this problem, restoration interventions have been implemented in a few selected areas since the 1970s, resulting in a mosaic of habitats, including a state forest (SF) restored through natural regeneration, and a community-managed forest (CMF) restored through natural regeneration and enrichment planting. Despite these limited efforts, land-use transformation continues into degraded shrubland (SL), with large areas converted to intensively cultivated cropland (CL)^3,42^. Using 16 S rRNA gene metabarcoding across five habitat types (CF, SF, CMF, SL, and CL), we examined patterns of soil prokaryotic turnover along a restoration–degradation gradient. We hypothesized that: (1) soil prokaryotic community composition and diversity would differ significantly among habitat types, without assuming a monotonic relationship between diversity and disturbance; (2) the variation in prokaryotic community composition and diversity would be associated with differences in soil properties, stand characteristics, and indicator woody species, reflecting shared environmental gradients rather than direct causal effects; (3) Indicators of community assembly processes would suggest a greater relative importance of deterministic processes in less-disturbed habitats (CF and SF), whereas stochastic processes would be more evident in disturbed habitats (CMF, SL, and CL); and (4) taxonomically inferred functional groups related to nitrogen cycling vary among habitats in parallel with shifts in community composition and assembly processes.

## Materials and methods

### Study area description

**Fig. 1:**
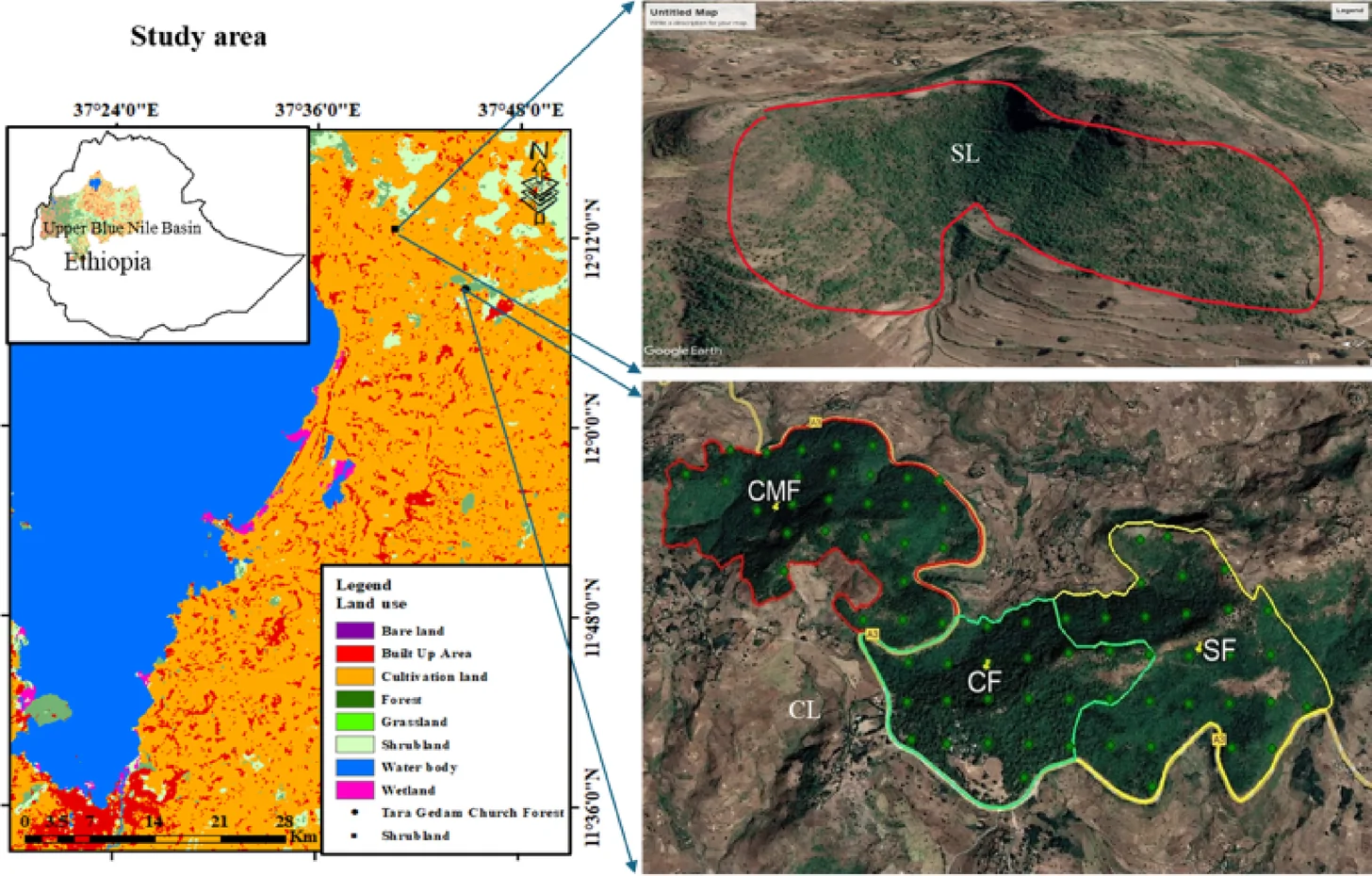
Location map of the study areas (left) and google earth views of different habitat types (right) in dry Afromontane Forest, northwest Ethiopia. Church forest (CF), state forest (SF), community managed forest (CMF), shrubland (SL), cropland (CL).

The study was conducted in Tara Gedam CF and adjacent habitats, including SF, CMF, SL, and CL, located in the dry Afromontane zone of northwest Ethiopia (12°8ʹ47″N, 37°44ʹ45″E; Fig. 1). This area lies at elevations ranging from 2062 to 2318 m and is classified as a dry subhumid Afromontane ecosystem, with an aridity index of 0.63^42^. The region experiences a prolonged dry season and receives an average annual rainfall of 1190 mm (June to September), with mean daily temperatures ranging from 13 to 27 °C (2000 to 2022) (Fig. S1: Adis Zemen meteorological station). The dominant soil type is nutrient-poor Cambisol with a loamy sand texture^42^. This is one of the densely populated regions of Ethiopia, and as a result, deforestation and agricultural expansion have altered the vegetation cover and soil conditions^41^.

The Tara Gedam landscape encompasses a mosaic of heterogeneous habitats characterized by varying vegetation compositions and disturbance histories (Fig. S2**)**. We stratified the landscape into five habitat types. The reference habitat, CF, represents a remnant DAF located within a Ethiopian Orthodox Tewahedo Church compound and is protected by local religious communities^4,5^. This forest exhibits the characteristics of a mature, climax ecosystem, dominated by native tree species typical of DAFs, including *Olea europaea*, *Albizia schimperiana*, *Teclea nobilis*, and *Juniperus procera*. These species are recognized for their ecological significance, providing structural stability, microhabitats, and nutrient cycling^1^. The dominance of these long-lived native species indicates minimal human disturbance and reflects the climax nature of CF, making it an ecological benchmark for assessing other habitat types. Restored habitats include SF, which was slightly disturbed in the past, with some large trees remaining, and has been restored through natural regeneration under local government management since the 1970s. The second restored habitat is CMF, which was heavily degraded owing to large tree removal and then restored via a combination of natural regeneration and enrichment planting with exotic species under the management of local communities. The degraded habitats consist of SL, which was converted from native forest and is now dominated by sparse shrubs and subjected to communal free grazing, and CL, which was also converted from native forest and is cultivated by smallholder farmers with annual crops such as *Eragrostis tef*, *Zea mays*, and *Vicia faba*, involving intensive tillage and associated soil disturbance^41^.

### Sampling methods

Permission for soil sampling was obtained from the Tara Gedam Monastery administration and the relevant local authorities prior to fieldwork. A total of 94 circular sampling plots (each 20 m in diameter) were established by using a systematic grid with 200-m spacing, in accordance with established protocols for vegetation and soil surveys^43^. Although this design may introduce spatial autocorrelation, such effects are scale-dependent and often reduced in heterogeneous forest ecosystems, where variability in vegetation and the soil environment disrupts spatial dependence^44,45^. Plots were distributed among habitat types primarily in proportion to their spatial extent within the study area: 22 in CF, 20 in SF, 20 in CMF, 12 in SL, and 20 in CL. This proportional allocation ensured representative coverage. Fewer plots in SL reflect its smaller area and more homogeneous vegetation, whereas larger, more heterogeneous habitats received more plots to improve estimate reliability. In each plot, all trees or shrubs, or both (diameter at breast height > 2 cm) were inventoried. Forest stand characteristics (stand density, basal area, and relative abundance of each species) were estimated for each plot. Before soil sampling, the organic horizon was manually removed. Undisturbed soil samples were taken by using cylindrical soil cores with a 5-cm diameter to determine bulk density (BD). Composite soil samples (at the plot center and at four points 5 m away in each cardinal direction) were then collected from 0 to 15 cm depth by using a ruler and hand shovel. Approximately 400 g of soil per plot was taken in plastic bags and transported to the Gondar Agricultural Research Center (Gondar, Ethiopia). After homogenization, about 2 g of soil (plant material removed) from each sample was preserved at − 20 °C (for no more than 20 days) for DNA extraction and later shipped to the Arid Land Research Center at Tottori University, Japan. The remaining soil was air-dried and processed for physicochemical analyses.

### Soil analysis

Soil pH and electrical conductivity (EC) were measured in a 1:2.5 soil: water suspension. Total organic carbon (TOC) and total nitrogen (TN) were measured by using the Walkley-Black and Kjeldahl methods, respectively. Exchangeable calcium (Ca²⁺), magnesium (Mg²⁺), potassium (K⁺), and sodium (Na⁺) were measured following ammonium acetate extraction and analyzed by using atomic absorption spectrophotometry. Soil BD was estimated from oven-dried soil cores (dried at 105 °C for 24 h) as the ratio of dry mass to soil volume.

### Soil DNA extraction, PCR amplification, and sequencing

Soil DNA was extracted from 0.25 g of homogenized soil sample by using a DNeasy PowerSoil Pro Kit (Qiagen, Hilden, Germany) in accordance with the manufacturer’s protocol. For prokaryotic community analysis, the V4 region of the 16 S rRNA gene was amplified by using the primers 515 F (GTGYCAGCMGCCGCGGTAA)^46^ and 806R (GGACTACNVGGGTWTCTAAT)^46^. Amplifications were performed on a Bio-Rad T100 thermal cycler (Bio Rad Laboratories, Hercules, CA) in a 25-µL PCR reaction mixture. PCR cycling conditions consisted of an initial denaturation at 98 °C for 30 s, followed by 30 cycles of denaturation at 98 °C for 10 s, annealing at 55 °C for 30 s, and extension at 72 °C for 1 min, with a final extension at 72 °C for 2 min. Barcoding was performed in a separate 25-µL reaction by using the same cycling conditions but with eight cycles. The quality of each PCR product was assessed by using 1.2% agarose gel electrophoresis. The products were purified by using Agencourt AMPure XP beads (Beckman Coulter, Inc., Brea, CA, USA) and quantified by using a Qubit dsDNA HS Assay Kit with a Qubit Fluorometer 2.0 (Invitrogen). Equimolar concentrations of purified DNA were pooled. Target gene regions were further purified by using agarose gel electrophoresis with a Monarch DNA Gel Extraction Kit (Monarch, NEB, USA), and fragment distribution and size were verified by using an Agilent 2100 Bioanalyzer (Agilent Technologies, Santa Clara, CA, USA). Next-generation sequencing was performed on an Illumina MiSeq platform (2 × 300 bp) by Macrogen Japan Co., Ltd (Tokyo, Japan) (ID: HN00206126), and the results were provided as FASTQ files.

### Bioinformatic analysis

Sequence data were preprocessed in R v4.3.2^47^ by using the DADA2 package (v1.30.0)^48^. Raw sequences were first trimmed to remove adapter and primer sequences by using the cutadapt plugin (v4.5)^49^. Quality assessment revealed substantially lower quality scores in reverse reads, particularly toward the 3′ ends. Forward and reverse reads were initially filtered using relaxed error thresholds (maxEE = c(2,5)); however, fewer than 50% of paired-end reads were retained after quality filtering and merging. To maximize retention of quality sequences and reduce sequencing artifacts, only forward reads were retained for amplicon sequence variant (ASVs) inference. Although single-end reads may reduce taxonomic resolution, high-quality forward reads provide reliable estimates of microbial diversity and community composition and are commonly applied in environmental microbiome studies when reverse-read quality is suboptimal^50–52^. Forward reads were filtered and trimmed by using the filterAndTrim function with the conditions of maxN = 0, maxEE = 2, truncQ = 2, truncLen = 240. Filtered sequences were subsequently dereplicated and denoised by using the dada function, which removes singletons, corrects sequencing errors, and infers exact ASVs. Chimeras were identified and removed by using the consensus method, and then a high-confidence ASV dataset for the prokaryotic community was retained^53^. Taxonomic assignment was performed by using SILVA (release 138.1)^54,55^ database for representative ASVs. To construct a phylogenetic tree, the ASV sequences were first aligned by using the DECIPHER package (v2.30.0)^56^. A neighbor-joining tree was then constructed, followed by the fitting of a generalized time-reversible model with gamma rate variation by using maximum likelihood implemented in the phangorn package v2.11.1^57^. ASVs identified as mitochondrial, chloroplast, or unclassified at the phylum level were excluded, retaining only those assigned to “Bacteria” and “Archaea”^58^. To standardize sequencing depth, samples were rarefied to 1148 denoised reads per sample using the mecodev package (v.0.2.0)^58^. This threshold was selected to maximize sample retention across habitats while minimizing the exclusion of biological replicates. Sampling completeness was evaluated using rarefaction curves based on observed ASVs, coverage, and Shannon diversity (Fig. S3). Coverage and Shannon diversity curves approached stabilization near the selected threshold, indicating adequate capture of dominant diversity patterns. In contrast, observed ASVs did not fully plateau, suggesting incomplete recovery of rare taxa, as commonly observed in microbial amplicon datasets^59^. To evaluate the robustness of ecological inference to sequencing depth, sensitivity analyses were conducted at rarefaction thresholds of 1148, 2000, and 3000 denoised reads per sample. Alpha diversity, beta diversity, PERMANOVA results, and betaNTI/RCBray-based community assembly inferences exhibited consistent ecological patterns across thresholds. Repeated rarefactions (*n* = 50) with averaging were additionally performed at 1148 reads per sample to assess stochastic variation introduced by subsampling on alpha diversity estimates^60^. Five samples with insufficient sequencing depth were excluded from downstream analyses, resulting in final rarefied sample sizes of 22 for CF, 20 for SF, 20 for CMF, 10 for SL, and 17 for CL. Putative functional groups were inferred using the Functional Annotation of Prokaryotic Taxa (FAPROTAX v1.2.6) database^40^ and interpreted as predicted functional potential. Sequence data have been deposited in the NCBI under the accession number PRJNA1294483.

### Statistical analysis

To identify characteristic tree or shrub species associated with each habitat, an indicator species analysis was conducted by using the indicspecies package v1.7.13^27^. To reduce multicollinearity among environmental predictors, Spearman’s rank correlations were computed for all variables. Variables showing strong pairwise correlations (|ρ| > 0.7) were considered collinear, and one variable from each correlated pair was excluded based on ecological relevance and redundancy, retaining a non-collinear subset for subsequent analyses^61^. Variance inflation factor (VIF) analysis further confirmed low multicollinearity among retained variables (Table S1). The retained variables were incorporated into a redundancy analysis (RDA). The significance of the overall model, individual explanatory variables, and canonical axes were assessed using Monte Carlo permutation tests (999 permutations). Environmental variables were then fitted onto the ordination using the envfit procedure, and their contributions were evaluated based on coefficients of determination (r²) and permutation-derived p-values. Variables with *p* < 0.05 were considered significant predictors. As most variables violated the assumptions of normality (Shapiro–Wilk test), the Kruskal–Wallis test with Dunn’s post hoc test (Holm-adjusted) was used to assess differences in stand and soil variables among habitats. The most abundant prokaryotic phyla were identified based on their relative abundances across all samples to determine the dominant microbial communities. Prokaryotic alpha diversity among habitats was evaluated using Shannon’s index and Faith’s phylogenetic diversity (PD) index, with significant differences tested by using the same non-parametric approach. To assess differences in microbial community structure among habitats, we performed PERMANOVA (999 permutations) by using Bray–Curtis, Jaccard, and weighted UniFrac distances^62^. Homogeneity of dispersion, an assumption of PERMANOVA, was tested using PERMDISP for each distance metric to ensure that significant results reflected true between-group differences^63^. Where significant differences were detected, pairwise comparisons were subsequently conducted with Benjamini false discovery rate (FDR) adjustment of P-values. Principal coordinate analysis was then applied to visualize the community structure among habitats. Linear discriminant analysis effect size (LEfSe) (linear discriminant analysis score ≥ 4.0, *P* ≤ 0.05, FDR adjusted) was used to identify habitat-specific differentially abundant taxa^64^. Redundancy analysis (RDA) was conducted at the phylum level to examine relationships between microbial communities and environmental variables, acknowledging reduced taxonomic resolution. Mantel tests (999 permutations) were also applied to assess the correlations between environmental matrices and microbial community dissimilarities at the ASV level^65^. Differences in dominant prokaryotic functional groups among habitats were evaluated by using the Kruskal–Wallis test to assess the impact of habitat degradation on the key functions that distinguish habitat conditions and contribute to nutrient cycling. Spearman’s correlation analysis was applied to examine the relationships among dominant phyla, microbial functions, and environmental variables.

To investigate niche-based selection, phylogenetic signal was assessed under the assumption that closely related taxa tend to share similar habitat preferences^38^. Mantel correlograms with Spearman correlations were used to assess correlations between phylogenetic distance and environmental niche differences^58^, thereby linking ASVs to environmental variables. We further assessed community assembly processes across rarefaction thresholds by integrating phylogenetic information (> 0.01% relative abundance) with the null model to infer the ecological relatedness between taxa on the basis of their phylogenetic similarity^33,36,38^. Specifically, we inferred the betaMNTD (beta mean nearest taxon distance) and derived the betaNTI (beta nearest taxon index) by comparing the observed values to a null distribution (1000 randomizations) of taxa across the phylogeny (taxa.labels model), which preserved both alpha and beta diversity. Following established thresholds^36,37^, a |betaNTI| value > 2 signified that deterministic selection was the dominant process: betaNTI values > + 2 indicated higher observed turnover than expected, owing to heterogeneous selection, whereas values < − 2 reflected lower observed turnover than expected, driven by homogeneous selection. For comparisons where |betaNTI| < 2, we assessed stochastic processes by using Raup-Crick Bray-Curtis dissimilarities (RCbray), preserving the species occurrence frequencies and sample richness^37^. The thresholds of RCbray > 0.95, < − 0.95, and |RCbray| < 0.95 indicated dispersal limitation, homogenizing dispersal, and ecological drift, respectively^34,36^. The relative contributions of deterministic and stochastic processes to community assembly were estimated on the basis of the proportions of betaNTI and RCbray values falling within these thresholds^36^. To evaluate the influence of habitat degradation on phylogenetic (betaNTI) and compositional (RCbray) turnovers, habitat-level differences in the betaNTI and RCbray values were compared by using the Kruskal–Wallis test with Dunn’s post hoc comparisons. All statistical analyses were conducted in R 4.3.2^47^ by using the microeco package (v1.4.0)^58^.

## Results

### Soil properties, stand structure, and indicator species

**Fig. 2:**
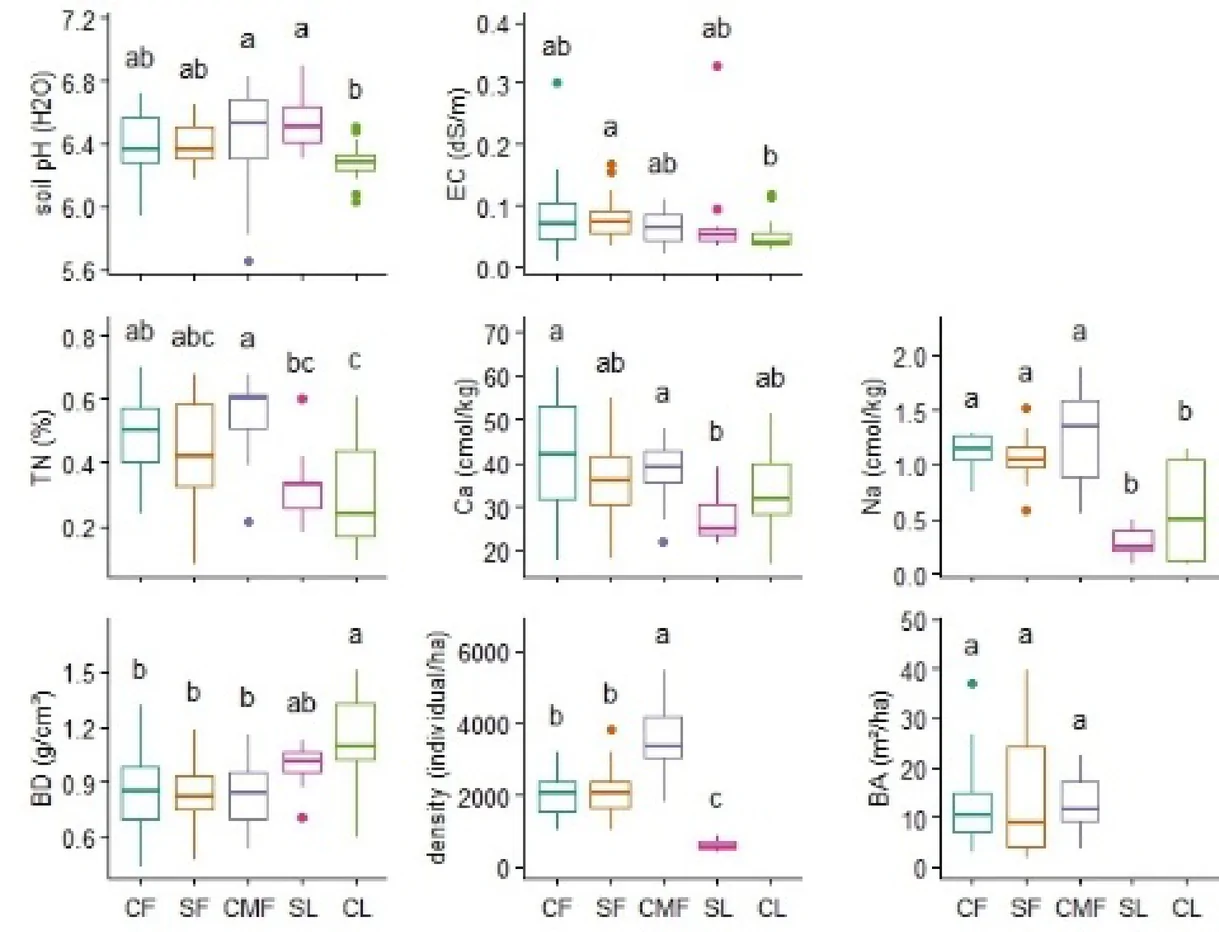
Soil properties and characteristics across different habitats. Different lowercase letters indicate significant differences between habitats (*P* < 0.05) on the basis of Dunn’s post hoc test following Kruskal–Wallis analysis. EC, electrical conductivity; TN, total nitrogen; Ca, exchangeable calcium; Na, exchangeable sodium; BD, bulk density; Density, tree/shrub stand density; BA, stand basal area.

Soil physicochemical properties and stand characteristics differed among habitat types. Soil pH was significantly higher (*P* < 0.05) in CMF and SL than in CL, whereas BD was significantly lower in CF, SF, and CMF than in CL (Fig. 2). EC was significantly higher in SF than in CL but showed no consistent pattern across habitats. TN tended to be high in CF and SF and was significantly higher in CMF than in SL and CL (Fig. 2). Exchangeable Na⁺ was significantly higher in CF, SF, and CMF than in in SL and CL, while Ca²⁺ was significantly higher in CF and CMF than in SL.

Indicator species analysis identified habitat-specific tree species (Table 1). *Teclea nobilis* and *Olea capensis* were associated with CF, whereas *Nuxia congesta*, *C. lusitanica*, and *Ocimum gratissimum* were linked to CMF and *M. arbutifolia* to SL. Some species were shared among habitats, including *Olea europaea* and *A. schimperiana* (CF and SF) and *Croton macrostachyus* (CMF and SL). Stand density was significantly higher in CMF than in CF, SF, and SL (*P* < 0.05), although basal area did not differ significantly among habitats (Fig. 2).

**Table 1:** Indicator tree and shrub species for each habitat, identified on the basis of indicator value analysis.

Species	Habitat types	Indicator value	*P* value
*Teclea nobilis*	CF	0.783	0.001
*Olea capensis*	CF	0.674	0.001
*Nuxia congesta*	CMF	0.639	0.005
*Cupressus lusitanica*	CMF	0.613	0.001
*Ocimum gratissimum*	CMF	0.612	0.001
*Maytenus arbutifolia*	SL	0.504	0.001
*Solanum gigantum*	CF + SF + SL	0.608	0.023
*Olea europaea*	CF + SF	0.598	0.015
*Albizia schimperiana*	CF + SF	0.734	0.001
*Croton macrostachyus*	CMF + SL	0.708	0.005
*Grewia ferruginea*	CMF + SF	0.668	0.001

### Soil prokaryotic community composition and diversity

**Fig. 3:**
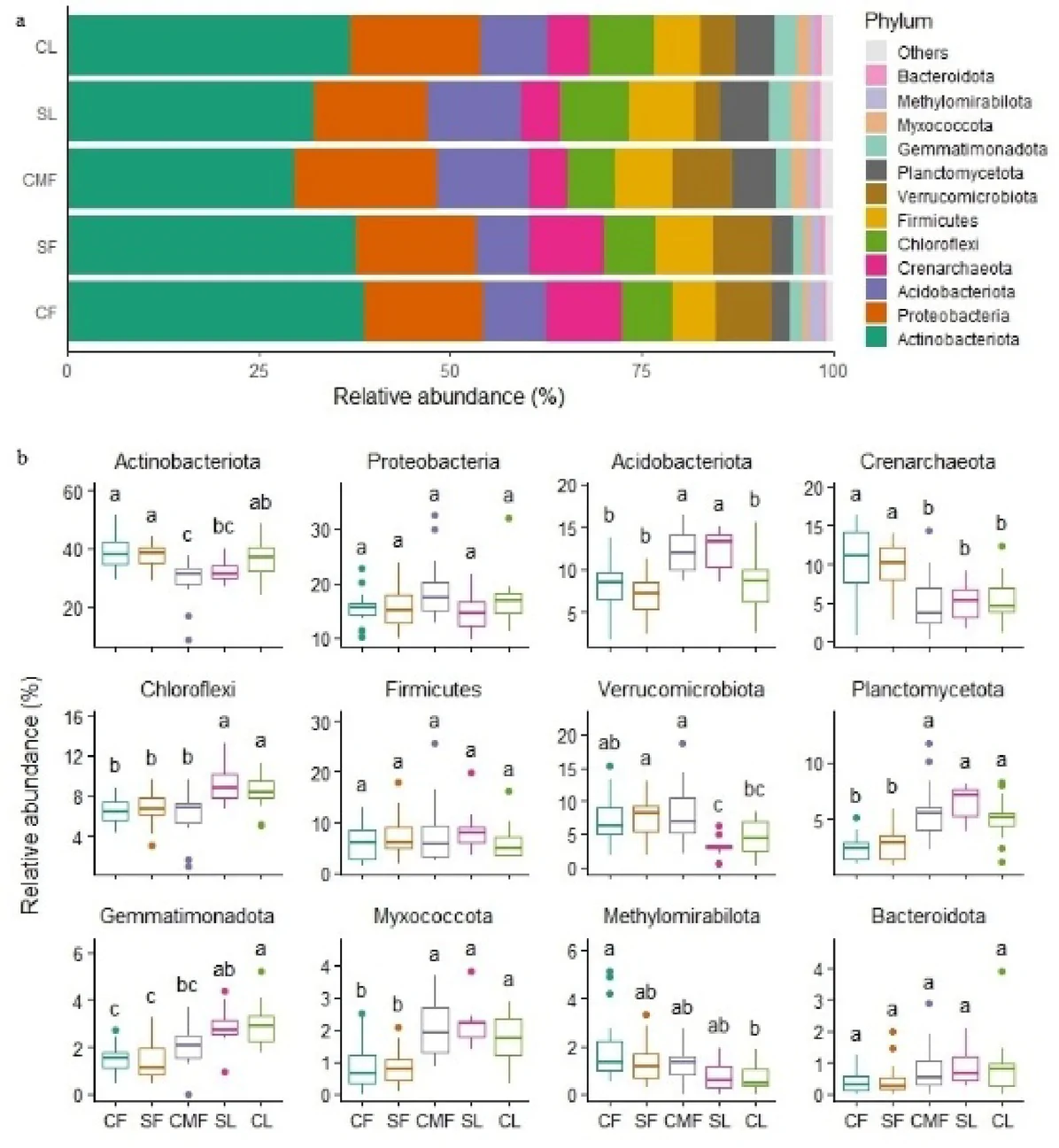
Relative abundances of predominant prokaryotic phyla **(a)** and their comparison **(b)** across habitats. Different letters indicate significant differences between habitats, based on Dunn’s post hoc following Kruskal–Wallis analysis (adjusted *P* < 0.05).

Soil prokaryotic community composition differed significantly among habitat types. Across all habitats, prokaryotic communities were dominated by Actinobacteriota (31.51%), Proteobacteria (14.67%), Acidobacteriota (8.32%), and Crenarchaeota (6.54%) (Fig. 3a). Their relative abundances varied significantly among habitats (Fig. 3b). Actinobacteriota were significantly more abundant in CF and SF than in CMF and SL, and Crenarchaeota were significantly more abundant in CF and SF than in CMF, SL, and CL (Fig. 3b). Verrucomicrobiota was significantly more abundant in CF, SF, and CMF than in SL and CL. In contrast, Acidobacteriota were significantly less abundant in CF and SF than in CMF and SL; Chloroflexi were significantly less abundant in CF, SF, and CMF than in CL and SL, and Planctomycetota and Myxococcota were significantly less abundant in CF and SF than in CMF, CL, and SL. Gemmatimonadota were significantly less abundant in CF, SF, and CMF than in CL. The relative abundances of Proteobacteria, Firmicutes, and Bacteroidota did not differ significantly among habitats.

**Fig. 4:**
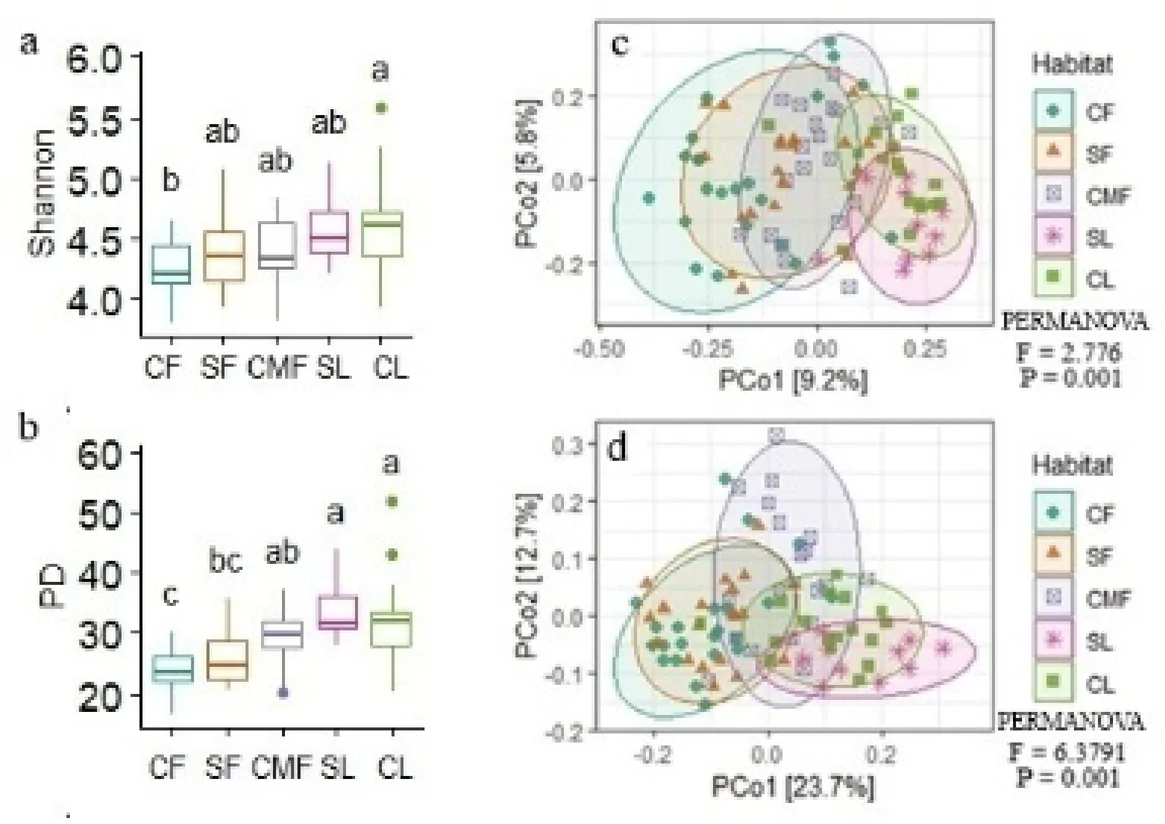
Alpha diversity and structure of the soil prokaryotic community among habitats. **(a)** Shannon’s and **(b)** Faith’s phylogenetic diversity (PD) indices; **(c)** principal coordinate analysis based on Bray–Curtis and **(d)** weighted UniFrac distances. Permutational analysis of multivariate dispersions (PERMDISP) indicated no significant differences in dispersion: Bray–Curtis, F = 0.5274, *P* = 0.725; Weighted UniFrac, F = 1.9173, *P* = 0.109.

The total ASVs varied across habitats (Fig. S6a), highest in CMF (1785), followed by CF (1628), CL (1615), SF (1538), and SL (1116). Unique ASVs showed a similar trend, with highest in CMF (1165), followed by CL (1027), CF (902), SF (866), and SL (729). Shared ASVs were low, with higher overlap between CF and SF (123) and CL and SL (92), while 121 ASVs were common to all habitats. Diversity indices also differed among habitats. The Shannon’s and Faith’s PD diversity indices of soil prokaryotes were generally lower in the undisturbed or restored habitats than in the degraded ones (Fig. 4a, b), although a significant difference in Shannon diversity was observed only between CF and CL. Faith’s PD in CF and SF was significantly lower than in SL and CL, and Faith’s PD was also significantly lower in CF than in CMF (Fig. 4b). No significant differences were found between CF and SF. Sensitivity analyses across rarefaction thresholds showed consistent alpha-diversity patterns (Fig. S4). Similarly, average Shannon diversity and Faith’s PD values derived from repeated rarefactions (*n* = 50 iterations) (Fig. S5) were consistent with results obtained from single rarefaction (Fig. 4a and b).

PERMANOVA revealed a significant difference in community composition among habitats based on Bray–Curtis, Jaccard, and weighted UniFrac distances (Fig. 4c, d, and S6b). PERMDISP detected no significant differences in dispersion (Bray–Curtis: F = 0.5274, *P* = 0.725; Jaccard: F = 1.7966, *P* = 0.126; weighted UniFrac: F = 1.9173, *P* = 0.109). Principal coordinate analysis further illustrated clear separation among habitats, with CF and SF clustering together and apart from SL and CL, with CMF positioned intermediately (Fig. 4c and d). Pairwise comparisons indicated significant differences among habitats, except between CF and SF (Table S2). These beta-diversity patterns and PERMANOVA results were consistent across rarefaction thresholds (Fig. S7, and Table S2). LEfSe analysis identified habitat-specific prokaryotic taxa (Fig. S8a). CF was enriched with Crenarchaeota and Nitrososphaeraceae-related groups, SF with Actinobacteriota and Thermoleophilia, CMF with Vicinamibacteria, SL with Acidobacteriota, Planctomycetota, and Chloroflexi, CL with Gammaproteobacteria and Gaiellales.

### Associations between environmental variables and prokaryotic composition

**Fig. 5:**
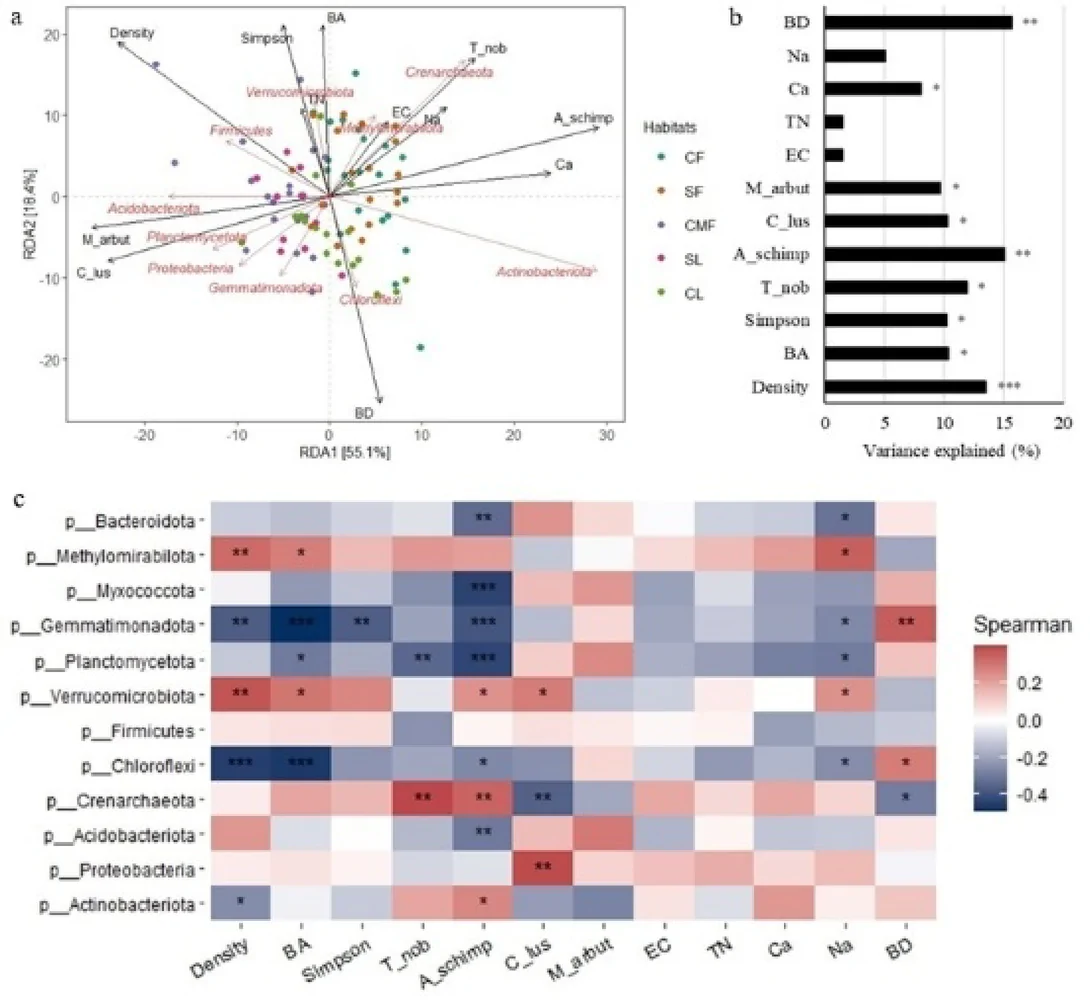
Constrained ordination analysis of prokaryotic communities in church forest and adjacent habitats. **(a)** Redundancy analysis (RDA) of prokaryotic communities in relation to soil properties and stand characteristics, showing the top 12 most abundant phyla; **(b)** the variance explained by each variable; and **(c)** Spearman’s correlations between the top 12 phyla and environmental factors. Asterisks indicate significance levels: *P* < 0.05 (*), *P* < 0.01 (**), and *P* < 0.001 (***). Abbreviations: *Teclea nobilis* (T_nob), *Albizia schimperiana* (A_schimp), *Cupressus lusitanica* (C_lus), *Maytenus arbutifolia* (M_arbut), stand density (Density), basal area (BA), electrical conductivity (EC), total nitrogen (TN), bulk density (BD), Simpson’s index (Simpson), church forest (CF), state forest (SF), community managed forest (CMF), shrubland (SL), cropland (CL).

Variation in prokaryotic composition was strongly associated with environmental gradients. Redundancy analysis (RDA) showed that first two axes explained 73.5% of the variation in the abundance of prokaryotic composition (Fig. 5a). Stand and soil variables, including BA, stand density, soil BD, and Ca²⁺ were significantly associated with community variation (Fig. 5b). Although their relative abundances were low, Verrucomicrobiota and Methylomirabilota were positively correlated with stand density, BA, and soil Na⁺, whereas Chloroflexi and Gemmatimonadota were negatively correlated with those variables but positively correlated with BD. Planctomycetota was negatively correlated with BA and Na⁺, and Actinobacteriota with stand density (Fig. 5c).

**Fig. 6:**
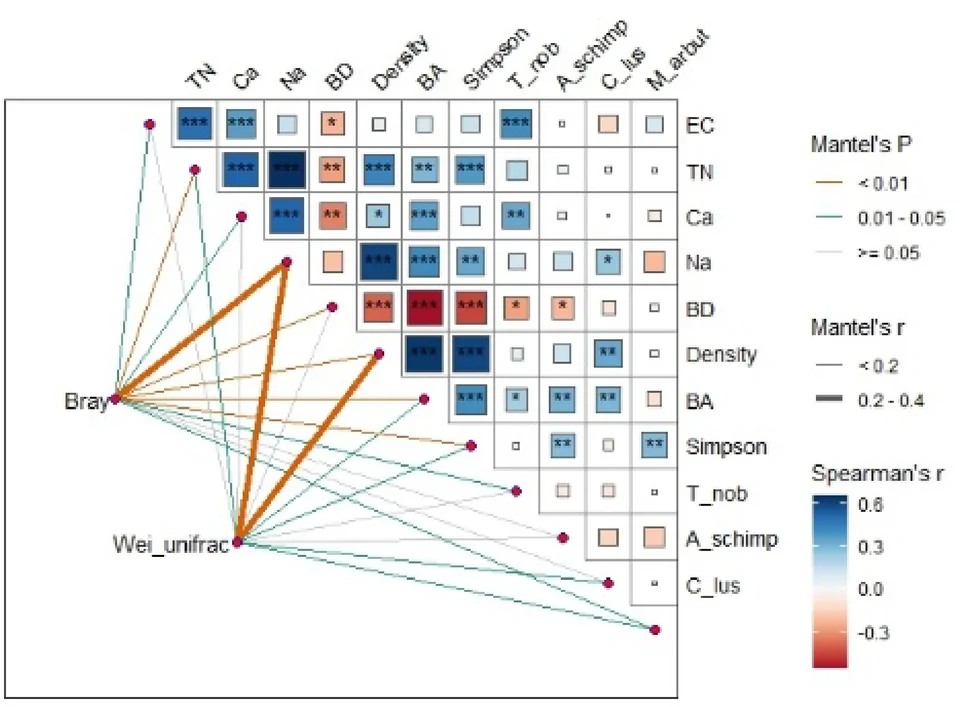
Mantel tests of the relationships between environmental factors and prokaryotic community structure (at the landscape scale). Analyses are based on Bray-Curtis (Bray) and abundance weighted UniFrac (Wei_unifrac) distance matrices. The color gradient shows spearman’s correlation coefficient between environmental factors, with significance levels indicated in each box (*P* < 0.05 (*), *P* < 0.01 (**), and *P* < 0.001 (***)). Edge width matches Mantel’s r statistic for the corresponding distance correlations, whereas edge color shows significance based on 999 permutations. Abbreviations: *Teclea nobilis* (T_nob), *Albizia schimperiana* (A_schimp), *Cupressus lusitanica* (C_lus), *Maytenus arbutifolia* (M_arbut), stand density (Density), basal area (BA), electrical conductivity (EC), total nitrogen (TN), bulk density (BD), Simpson’s index (Simpson).

Furthermore, indicator tree species were also significantly associated with variation in community composition (Fig. 5b). The indicator trees (*T. nobilis*,* A. schimperiana*,* C. lusitanica*, and *M. arbutifolia*) showed significant associations with dominant phyla (Fig. 5b). Actinobacteriota and Crenarchaeota were positively associated with *A. schimperiana*, with Crenarchaeota also positively correlated with *T. nobilis*, but negatively to *C. lusitanica*. In contrast, Proteobacteria and Verrucomicrobiota were correlated positively with *C. lusitanica*. Several other phyla showed negative association with *A. schimperiana*, some also negatively related with *T. nobilis*. The relationships between the Bray–Curtis and weighted UniFrac dissimilarities and environmental factors were assessed by using the Mantel test (Fig. 6). Both metrics showed strong correlations with stand density, BA, TN, and Na⁺. Additionally, EC, Ca^2+^, and BD were significantly correlated with the Bray–Curtis distance. Among all variables, stand density and soil Na⁺ were strongly correlated with both distance matrices. Indicator tree species showed metric-specific associations, while *A. schimperiana* showed no significant correlation.

### Prokaryotic community assembly

**Fig. 7:**
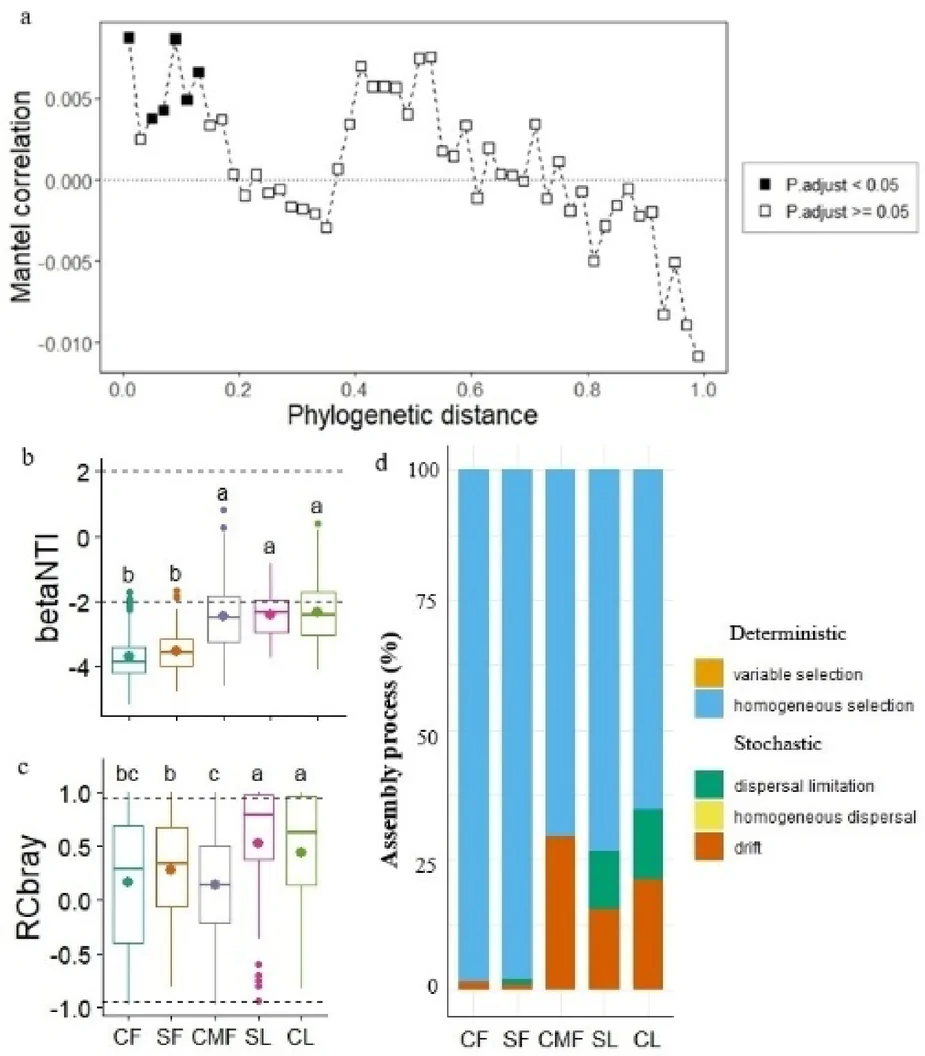
Soil prokaryotic community assembly mechanisms across church forest (CF) and adjacent habitats. **(a)** Mantel correlogram shows the phylogenetic signal based on correlations between environmental (niche) and phylogenetic distances among amplicon sequence variants. Distributions and comparisons of phylogenetic (betaNTI) **(b)** and compositional (RCbray) **(c)** turnovers across different habitat types. **(d)** Relative contributions of community assembly processes within each habitat. State forest (SF), community managed forest (CMF), shrubland (SL), cropland (CL).

Significant phylogenetic signals (*P* < 0.05) were observed across short phylogenetic distances (Fig. 7a). BetaNTI values ranged mostly from 0 to − 5 (|betaNTI| > 2). Mean betaNTI values were significantly lower in CF and SF than in CMF, SL, and CL (Fig. 7b). Similarly, RCbray values were significantly lower in CF, SF, and CMF than in SL and CL (Fig. 7c). Partitioning of assembly processes showed that homogeneous selection accounted for 81.1% of total community turnover (Fig. S9) and its contribution was highest in CF (98.3%) and SF (97.3%) compared with CMF (70.5%), SL (73.3%), and CL (65.4%) (Fig. 7d). Among the stochastic processes, drift (13.8%) and dispersal limitation (5.1%) contributed to smaller proportion (Fig. S9), primarily in CMF, SL, and CL (Fig. 7d). Sensitivity analyses across rarefaction thresholds further showed consistent betaNTI, RCBray, and community assembly patterns (Figs. S10 and S11).

### Predicted functional potential and their correlations with soil properties and indicator trees

**Fig. 8:**
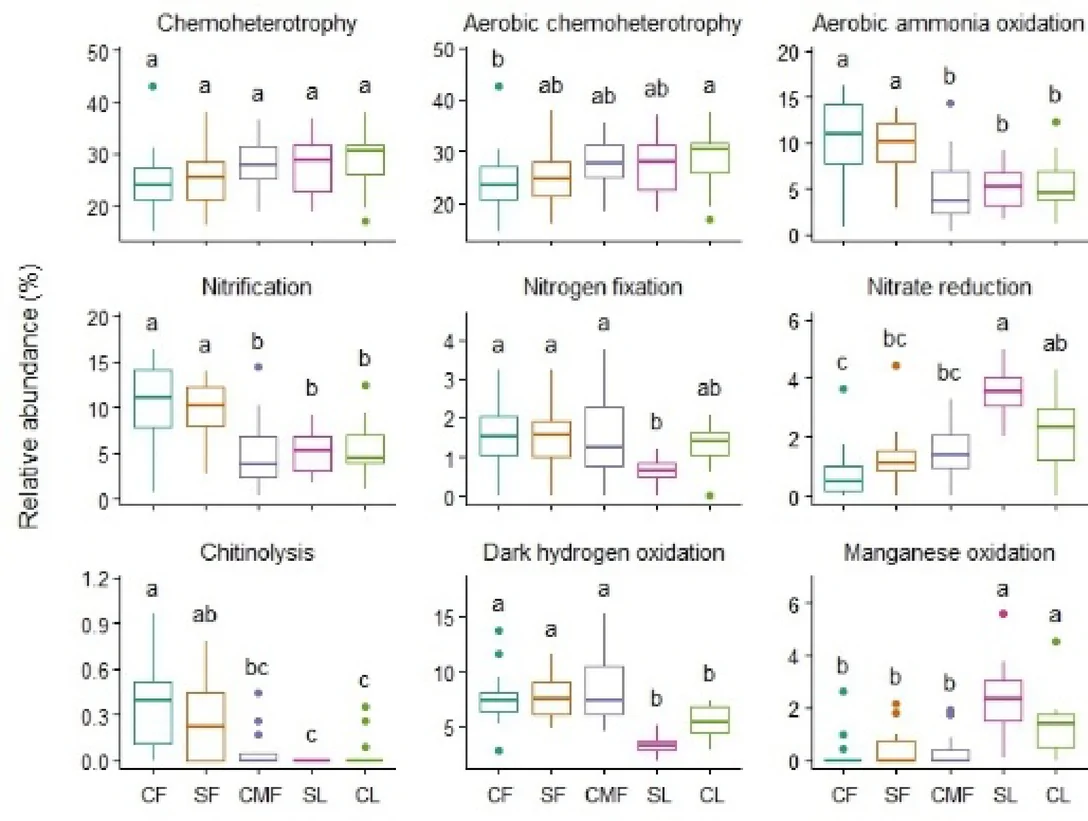
Relative abundances of dominant predicted functions across church forest (CF) and adjacent habitats. Different letters show significant differences in predicted functional groups between habitats based on Dunn’s post hoc following Kruskal–Wallis analysis (adjusted *P* < 0.05). State forest (SF), community managed forest (CMF), shrubland (SL), cropland (CL).

FAPROTAX assigned ASVs to 46 putative functional groups (Fig. S12). Across all habitats, chemoheterotrophy and aerobic chemoheterotrophy were the most abundant predicted functions (Fig. S13). The relative abundance of nitrogen-cycling functional groups, namely aerobic ammonia oxidation and nitrification, was significantly higher in CF and SF than in CMF, SL, and CL (Fig. 8) and positively correlated with *T. nobilis* and *A. schimperiana* (Fig. S14). Nitrogen fixation groups were also more abundant in CF, SF, and CMF than in SL. Predicted chitinolysis was more abundant in CF and SF than in SL and CL and significantly more abundant in CF than in CMF; this group was positively associated with the abovementioned indicator trees and with BA (Fig. S14). ASVs assigned to dark hydrogen oxidation were significantly more abundant in CF, SF, and CMF than in SL and CL. In contrast, manganese (Mn) oxidation were significantly less abundant in CF, SF, and CMF than in SL and CL, and those assigned to nitrate reduction were significantly less abundant in CF and SF than in SL (Fig. 8); these predicted functions were positively correlated with soil BD but were negatively correlated with Na⁺, Ca²⁺, stand density, BA, and *T. nobilis* (Fig. S14).

## Discussion

### Variations in stand characteristics and soil properties across CF and adjacent habitats

Differences in stand structure and soil properties were evident across habitat types, reflecting the restoration–degradation gradient in DAFs. These substantial differences in stand structure and soil properties have been associated with degradation and conversion of DAFs in northwest Ethiopia^5,66^. In this study, CF, SF, and CMF showed higher stand density (Fig. 2), aligning with previous studies^3,42^. Indicator species analysis confirmed that native tree species were characteristic of CF and SF but were largely absent in CMF, SL, and CL (Table 1). The prevalence of long-lived native species (*O. europaea* and *A. schimperiana*) in CF and SF is consistent with the structural complexity typically observed in less disturbed forests^1^. In contrast, enrichment planting of exotic species such as *C. lusitanica* in CMF was associated with increased stand density but altered species composition^67^. Similar vegetation shifts have been reported across dryland and tropical forest ecosystems, where variation in vegetation structure, litter inputs, and belowground habitat conditions have been associated with land-use conversion and restoration practices^10,13,42^.

Moreover, these vegetation differences are also associated with variations in soil properties^1^. CF, SF, and CMF generally exhibited higher nutrient levels and more favorable physical conditions than in SL and CL (Fig. 2). In particular, higher TN and exchangeable Na⁺ and Ca²⁺ concentrations, together with lower soil BD in CF and SF compared with SL and CL, were associated with higher vegetation cover and greater organic matter accumulation^42^. The relatively high Na⁺ in CF and restored habitats may also reflect to higher cation exchange capacity, and vegetation–soil association reported in tropical systems^68–70^. In addition, the traits of indicator species such as nitrogen fixation capacity and litter quality, may also contribute to variation in soil cation availability^71^. Comparable associations between vegetation cover, nutrient retention, and soil structure have been widely documented in global dryland ecosystems, where intact forest or shrub cover is commonly associated with greater organic matter accumulation and lower levels of soil degradation^10,19,72^. In contrast, degraded habitats (SL and CL) showed lower nutrient levels and higher soil compaction, patterns often associated with cultivation and grazing, which can reduce exchangeable cations and alter soil structure^72^. Similar declines in soil fertility and increases in compaction under disturbance have been reported across tropical dry forests, savannas, and Mediterranean drylands^10,19,73^. These patterns suggest that differences in vegetation structure and land use are closely associated with variation in soil properties across habitats. Such changes are likely associated with variation in soil microbial community structure and function, underlining the need for investigation into microbial responses to forest degradation and restoration.

### Prokaryotic community composition, diversity, structure, and drivers

Prokaryotic community composition, diversity, and structure in the studied DAF are strongly associated with environmental gradients across habitats, supporting our hypothesis that microbial communities differ significantly among habitat types. These patterns are consistent with studies from dryland and forest ecosystems showing that land-use change, vegetation loss, soil compaction, and nutrient depletion are associated with shifts in microbial community composition and ecosystem functioning^74–76^. The dominance of Actinobacteriota, Proteobacteria, Acidobacteriota, and Crenarchaeota across all habitats is consistent with the patterns in tropical and sub-Saharan Afromontane forest soils^10,17,77,78^. These phyla are known for their broad functional capabilities and ecological versatility^12^, although their relative abundances differed among habitats. CF and SF were characterized by higher relative abundances of Actinobacteriota and Crenarchaeota, consistent with undisturbed ecosystems where native vegetation may associated with a greater representation of niche specialist taxa^8,15,78^. Similar enrichment of specialist taxa in intact forests has been documented in tropical dry forests, and semi-arid ecosystems^10,17,77^. By contrast, the degraded habitats (SL and CL) showed greater representation of Chloroflexi, Planctomycetota, and Gemmatimonadota taxa commonly associated with disturbed or nutrient-limited soils^18,19,79^. Such enrichment of stress-tolerant taxa under degraded conditions is consistent with patterns reported in dryland, agricultural, and disturbed forest ecosystems^8,19,75,76,80^.

Most ASVs were unique to individual habitats (Fig. S6 a), with limited overlap among sites, and the small core community (121 ASVs) indicating strong habitat-specific structuring under land-use change^16,17,24^. Prokaryotic diversity in DAF soils appear comparable with some studies^81^ but lower than others^17,35,82^, These are likely to reflect the combined effects of strong environmental filtering, historical disturbance, and dispersal limitations associated with fragmentation^17,31,34,36^. However, the relatively low sequencing depth and use of single end reads likely reduced detection of rare taxa and total richness, although rarefaction curves approached stabilization for alpha diversity indices. Nevertheless, sensitivity analyses and repeated rarefication showed that alpha diversity remained stable for undersampling (Fig. S4, and S5), indicating that rarefaction at 1148 reads per sample captured the dominant community. Notably, Shannon’s index and Faith’s PD were lower in CF and SF than in SL and CL (Fig. 4a and b), supporting our hypothesis that diversity differs among habitats but does not follow a monotonic relationship with disturbance. Instead, diversity patterns appeared context-dependent, as reported previously^10,12,81,82^. Lower diversity in less disturbed forests may reflect stronger environmental filtering and competitive dominance, where a subset of habitat-specialist taxa prevail and limit overall richness^10^. In contrast, higher diversity in degraded habitats may be associated with increased environmental heterogeneity, suggesting the coexistence of taxa with broader ecological tolerances and reducing competitive exclusion^20,80,83^. Similar patterns have been reported across global environmental gradients^12^, including dryland forests where moderate disturbance or increased heterogeneity was associated with higher bacterial alpha diversity despite reduced ecosystem integrity^10,11,20^. Studies from tropical dry forests and semi-arid restoration systems likewise showed that degraded habitats supported more taxonomically diverse but functionally altered microbial assemblages dominated by stress-tolerant taxa^19,42,83^. Accordingly, higher alpha diversity in degraded habitats likely reflects community reorganization under modified environmental conditions rather than enhanced ecosystem stability. These results emphasize that alpha diversity alone provides a limited indicator of land-use impacts and should be interpreted alongside community structure, and community assembly processes when assessing microbial responses to degradation and restoration.

Community structure analyses (PERMANOVA, ordination) further confirmed strong habitat effects, with CF and SF forming clusters distinct from SL and CL, and CMF occupying an intermediate position (Fig. 4c and d). Despite the limited sequencing depth, the consistency of clustering patterns and statistical significance across rarefication thresholds supports the robustness of these differences (Fig. S7, and Table S2). Similar compositional separation between intact and degraded habitats has been observed across tropical forests, savannas, and global dryland ecosystems undergoing land-use change^18,19,76,79^. This pattern reinforces our hypothesis that habitat type exerts a strong influence on microbial community composition. It also aligns with evidence that soil microbiomes are highly sensitive to environmental change^14^ and that community shifts are often associated with selective enrichment or loss of habitat-specific taxa^84^. Differentially abundant taxa identified by LEfSe further highlighted habitat-associated patterns, with Crenarchaeota and Actinobacteriota in CF and SF (Fig. S8a), whereas stress-tolerant taxa (Acidobacteriota, Planctomycetota, Chloroflexi) were more associated with degraded habitats. These patterns were also correlated with variation in soil properties and forest characteristics, including exchangeable Na⁺, bulk density, and stand density **(**Fig. 5, S8b**)**.

Associations between microbial communities and environmental variables were also evident. Among soil properties, exchangeable Na⁺ showed strong correlations with taxonomic and phylogenetic structure (Fig. 6) and with the phyla Verrucomicrobiota and Methylomirabilota (Fig. 5c). These patterns indicate that variation in Na⁺ was associated with habitat-related differences in microbial composition and habitat condition, consistent with studies from dryland ecosystems under land-use gradients^85^. Similarly, exchangeable Ca²⁺ and TN were also associated with community variation, potentially reflecting their roles in soil chemistry and nutrient availability^28,86^. Similar associations between edaphic properties and microbial composition have been reported in dryland soils, where cation availability and soil physicochemical conditions are strongly correlated with microbial community organization^24,87^. The phyla Chloroflexi and Gemmatimonadota, which dominated in degraded soils (Fig. 3b) were negatively associated with Na⁺ but positively correlated with BD, consistent with their adaptation to compacted, nutrient-poor conditions^10,18^. Similar enrichment of these taxa in degraded drylands and disturbed forest soils has been documented^19,79^.

Stand characteristics—particularly stand density and BA— were correlated with shifts in prokaryotic community composition (Fig. 5a–c). Certain taxa (e.g., Verrucomicrobiota and Methylomirabilota) positively associated with denser stands, and others (e.g., Chloroflexi and Gemmatimonadota) more associated with higher bulk density and lower nutrient conditions. This indicates the role of forest structure in promoting or suppressing specific microbial groups^25,26^. The Mantel tests confirmed these findings, with stand density explaining the greatest variation in both Bray–Curtis and UniFrac dissimilarities (Fig. 6). Similar vegetation–microbiome relationships have been observed in forest and dryland ecosystems, where plant structural complexity and litter quality are associated with differences in microbial community composition through variation in soil organic matter, and nutrient inputs^24,88,89^. Indicator tree species were also associated with differences in prokaryotic composition: Native species (*A. schimperiana*, *T. nobilis*) were associated with taxa more abundant in less disturbed habitats, whereas disturbance-associated species (e.g., *C. lusitanica*) showed different associations. These patterns support broader evidence that vegetation type is associated with variation in soil microbial communities through litter inputs, and microhabitat modification^8,77,90,91^. However, these relationships are based on correlative analyses and should not be interpreted as evidence of direct causal effects. In addition, the redundancy analysis (RDA) was conducted at the phylum level, which provides a relatively coarse resolution and may obscure finer-scale taxonomic and functional relationships. Future studies using higher-resolution taxonomic data and experimental approaches would help to better resolve these associations. Overall, habitat degradation in DAFs is associated with shifts in prokaryotic community composition, characterized by increased representation of taxa commonly found in disturbed or nutrient-limited environments and reduced representation of taxa associated with less disturbed forest conditions.

### Prokaryotic community assembly processes across CF and adjacent habitats

Significant phylogenetic signals (Fig. 7a) suggest niche conservatism and support the use of phylogenetic approaches to examine community assembly mechanisms^36^. The widespread occurrence of |betaNTI| > 2 across all habitats further indicates the overall dominance of deterministic processes. In relatively stable habitats such as CF and SF, the lower phylogenetic turnover (Fig. 7b) indicates stronger environmental filtering under relatively consistent environmental conditions^87,92^. Similar dominance of deterministic assembly under stable conditions have been reported in dryland and forest ecosystems^32,87,92^. In contrast, the higher phylogenetic turnover in CMF, SL, and CL may reflect greater environmental heterogeneity associated with disturbance^35^. Comparable increases in stochastic assembly under degraded or managed conditions have been observed in agricultural transitions, reforestation systems, and disturbed dryland ecosystems^34,93,94^. Similarly, higher compositional turnover (RC_bray) in these habitats (Fig. 7c) is consistent with reduced homogeneous selection and greater compositional variability under disturbance^87^.

Deterministic processes, particularly homogeneous selection, accounted for a large proportion (81%) of the overall community turnover (Fig. S9), highlighting selective pressures as the dominant assembly mechanism. This finding is consistent with growing evidence from dryland and forest microbiome studies showing that microbial assembly is strongly associated with gradients of disturbance, vegetation change, and soil conditions^12,17,75,95^. Fine-scale studies have also associated vegetation structure and soil properties with microbial community assembly across ecosystems^36,87,92,93^. Habitat degradation and disturbance were associated with lower contributions of deterministic processes and greater stochastic influence^35,96^, a pattern increasingly recognized across restoration and disturbance gradients^31^. The relatively lower contribution of homogeneous selection in CMF (70.5%), SL (73.3%), and CL (65.4%) than in CF (98.3%) and SF (97.3%) (Fig. 7d) may be associated with differences in vegetation cover, soil condition, and organic matter inputs among habitats^25,26,32,97^. Similar reductions in deterministic assembly following vegetation loss and soil degradation have been reported in dryland restoration systems and disturbed forest ecosystems^32,93,94^. The occurrence of legume trees such as *A. schimperiana* as an indicator species in CF and SF may also contribute to habitat-related differences in microbial composition through litter and nutrient inputs^15^. In addition, stochastic processes, including ecological drift and dispersal limitation, contributed in CMF, SL, and CL (Fig. 7d), indicating mixed assembly patterns in disturbed or managed ecosystems^94,98^. These findings are consistent with succession frameworks in which stochastic influences become more prominent under variable environmental conditions, whereas deterministic filtering strengthens in relatively stable habitats^92,99^. The intermediate assembly patterns observed in a restored forest habitat (CMF) suggest partial convergence toward less disturbed habitats, although microbial composition and assembly patterns remained distinct from intact forests, consistent with previous restoration studies^13,42^. However, these inferences are based on null models and should be interpreted cautiously. Shallow sequencing depth may also bias estimates by underrepresenting rare taxa and possible stochastic detection^37^. Nevertheless, the consistent trends in betaNTI/RCBray-based assembly inference across rarefaction depths (Fig. S10 and S11) indicate that the dominance of homogeneous selection was robust to undersampling. Overall, deterministic assembly remained dominant across habitats, while additional stochastic influence along the disturbance and restoration gradient were consistent with reorganization of dryland microbiomes under land-use change.

### Predicted soil prokaryotic functional potential in CF and adjacent habitats

Habitat degradation was associated with pronounced shifts in the relative abundance of taxonomically inferred functional groups of soil prokaryotes, particularly those related to nitrogen cycling (Fig. 8). Shifts in microbial community composition under altered environmental conditions have been widely reported as sensitive indicators of ecosystem modification^14^. In relatively undisturbed habitats (CF and SF), predicted functional profiles showed higher relative abundance of ASVs assigned to aerobic ammonia oxidation and nitrification, indicating a greater representation of taxa commonly associated with these processes^100^. These patterns coincided with higher abundances of Crenarchaeota and Nitrososphaeraceae (Fig. S8b) and with indicator trees such as *T. nobilis* and *A. schimperiana* (Fig. S14). Similar enrichment of nitrification-associated taxa in relatively intact forests has been reported in tropical and dryland ecosystems, where stable vegetation cover and organic matter inputs were associated with higher relative abundances of nutrient-transforming microbial groups^77,81,100^. Predicted dark hydrogen oxidation was also more abundant in less disturbed habitats (Fig. 8), paralleling higher representation of putative nitrogen-fixing taxa. Such co-occurrence patterns may reflect overlapping ecological niches^101,102^. In contrast, ASVs assigned to nitrate reduction and manganese oxidation were relatively more abundant in the degraded SL and CL habitats (Fig. 8), consistent with altered soil conditions associated with greater representation of disturbance-adapted taxa^71,73,79,103^. Similar functional shifts toward stress-tolerant and oligotrophic microbial groups have been documented across degraded drylands, tropical dry forests, and semi-arid agricultural systems^10,19,103^. Among carbon-related functions, cellulolysis was widespread across all habitats (Fig. S13), reflecting its broad taxonomic distribution and central role in organic matter decomposition^104^. In contrast, the predicted chitinolysis was more abundant in CF and SF (Fig. 8) and was positively correlated with stand structure (Fig. S14). This pattern likely reflects differences in organic matter inputs and habitat structure rather than enhanced functional activity^105,106^. Comparable associations between forest structural complexity and decomposers have been reported in restored and intact forest ecosystems, where forest structural complexity was positively associated with predicted decomposition-related microbial functions^13,89^.

Overall, habitat degradation in DAFs is associated with shifts in the prokaryotic taxa linked to different metabolic potentials, from those associated with nutrient transformation in the less disturbed forests to those characteristics of degraded environment. Similar functional shifts have been documented across dryland and tropical forest microbiomes, where vegetation degradation was frequently associated with reduced relative abundance of taxa linked to nutrient cycling while increasing representation of stress-tolerant groups^107,108^. Studies from African savannas, Mediterranean ecosystems, tropical dry forests, and forest restoration chronosequences have likewise reported disturbance-linked transitions in nitrogen transformation pathways and decomposition-related microbial functions^13,73,103^. These convergent patterns suggest that environmental filtering associated with vegetation loss and soil degradation may be correlated to consistent patterns of functional reorganization across dryland forest microbiomes. However, functional predictions based on 16 S rRNA data represent potential rather than actual activity, and trait variability within taxa may limit inference accuracy^40,108,109^. Therefore, results should be interpreted cautiously given the shallow sequencing depth and reliance on inferred functions and require validation using metagenomic or process-based approaches. Together, these results indicate that habitat degradation is associated with coordinated changes in soil conditions, microbial community structure, and assembly processes. Conservation of intact forests and restoration of degraded habitats were associated with microbial communities characterized by greater representation of nutrient-cycling taxa and stronger deterministic assembly patterns in DAFs. More broadly, this study contributes to growing global evidence that dryland forest degradation is consistently associated with shifts in microbial community structure, assembly mechanisms, and predicted ecosystem functional potential across distinct biomes, highlighting the importance of incorporating belowground community into forest conservation and restoration strategies.

## Conclusions

Habitat degradation is associated with the shifts in soil prokaryotic communities in DAFs, reflected in changes in taxonomic composition, alpha diversity, and community assembly processes. Less disturbed habitats (CF and SF) were characterized by distinct community composition, including higher relative abundances of Actinobacteriota and Crenarchaeota, whereas degraded habitats showed increased representation of Acidobacteriota, Chloroflexi, and Planctomycetota. Prokaryotic alpha diversity was lower in CF and SF, consistent with strong environmental filtering rather than reduced ecological integrity. Microbial community patterns were closely associated with stand attributes and soil properties—particularly stand density, indicator trees, exchangeable Na^+^, and TN—highlighting importance of fine-scale habitat conditions. Community assembly was primarily driven by deterministic homogeneous selection across all habitats, most strongly in CF and SF, indicating strong niche-based filtering. Stochastic influences were also evident in CMF, SL, and CL, suggesting that restoration and disturbance induced variable conditions that allowed both environmental filtering and random processes. Taxonomically inferred functional profiles suggested shifts in prokaryotic groups associated with nitrogen cycling and stress-tolerance along the disturbance gradient. However, as the analyses are correlative and functions are inferred, the results do not establish causal relationships or direct ecosystem processes. Overall, our findings demonstrate that soil prokaryotic communities are sensitive to habitat modification and may serve as indicators of environmental change. From a management perspective, conserving intact forests and restoration interventions that enhance vegetation structure and soil quality may help maintain soil microbial community structure and potential ecosystem functioning. Future studies integrating direct functional measurements are needed to link microbial dynamics with ecosystem processes in dry Afromontane landscapes.

## Supplementary Information

Below is the link to the electronic supplementary material.


Supplementary Material 1


## Data Availability

The datasets generated and/or analysed during the current study are available in the NCBI repository, accession number PRJNA1294483.
