# Supplementary material for: Comparative analysis of soil prokaryotic community structure and function in Ethiopian Church Forests and adjacent habitats
Source: Sci Rep. 2026 Jun 11;16:27337. doi: 10.1038/s41598-026-57528-9 (PMC13529787; doi:10.1038/s41598-026-57528-9)
Supplement: Supplementary file 1 [file 41598_2026_57528_MOESM1_ESM.docx]

**Scientific Reports**

**Supplementary Information**

**Comparative analysis of soil prokaryotic community structure and function in Ethiopian Church Forests and adjacent habitats**

Biazen Endalamaw ^1, 2*^, Nigussie Haregeweyn ^3^, Takeshi Taniguchi ^3^, Getu Abebe ^2^, Masataka Nakayama ^4^, Mitsuru Tsubo ^3^, Menale Wondie ^2^, Takeshi Abe ^3^, Yoseph Buta Hailu ^1, 5^, Atsushi Tsunekawa ^3^

^1^ *The United Graduate School of Agricultural Sciences, Tottori University, 4-101 Koyama-Minami, Tottori 680-8553, Japan*

^2^ *Amhara Agricultural Research Institute, Forestry Research Department, P.O. Box 527 Bahir Dar, Ethiopia*

^3^ *International Platform for Dryland Research and Education, Tottori University, 1390 Hamasaka, Tottori 680-0001, Japan*

^4^ *Faculty of Environmental, Life, Natural Science and Technology, Okayama University, Okayama, Japan*

^5^ *School of Civil and Water Resources Engineering, Debre Markos Institute of Technology, Debre Markos University, P.O. Box 269 Debre Markos, Ethiopia*

^*^ Corresponding author. Email Adress: biazengarc@gmail.com

**Table**

**Table S1:** Variance inflation factor (VIF) values for environmental variables retained for downstream statistical analyses after multicollinearity screening.

| Number | Predictors | Variance Inflation Factor (VIF) |
| --- | --- | --- |
| 1 | pH | 1.446793 |
| 2 | EC | 2.178928 |
| 3 | TN | 2.982003 |
| 4 | avP | 1.798516 |
| 5 | BD | 1.918308 |
| 6 | Ca.. | 2.242759 |
| 7 | K. | 1.315232 |
| 8 | Na. | 3.368089 |
| 9 | Mg. | 1.333227 |
| 10 | Simpson | 2.980446 |
| 11 | Density | 3.279700 |
| 12 | BA | 2.979892 |
| 13 | A_schimp | 1.631755 |
| 14 | T_nob | 1.372530 |
| 15 | C_lus | 1.680070 |
| 16 | M_arbut | 1.627065 |

Note: Electrical conductivity (EC); Total nitrogen (TN); Available phosphorus (avP); Bulk density (BD); Basal area (BA), *Teclea nobilis* (T_nob), *Albizia schimperiana* (A_schimp), *Cupressus lusitanica* (C_lus), *Maytenus arbutifolia* (M_arbut).

**Table S2** Pairwise comparisons of habitats in dissimilarities of soil prokaryotic community across different rarefaction thresholds (1148, 2000, and 3000 denoised reads per sample). Analyses were performed following multivariate permutational analysis of variance (PERMANOVA) based on Bray–Curtis, and weighted UniFrac distances. Results are presented as pseudo-F ratios (F) with Benjamini FDR adjusted p-values for multiple comparisons

| Measures | Groups/Habitats/ | Rarefaction at 1148 reads (used in this manuscript) | | Rarefaction at 2000 reads | | Rarefaction at 3000 reads | |
| --- | --- | --- | --- | --- | --- | --- | --- |
|  |  | F | Adjusted P | F | Adjusted P | F | Adjusted P |
| Bray-Curtis | CF vs SF | 1.369 | 0.05 | 1.378 | 0.04 | 1.440 | 0.03 |
|  | CF vs CMF | 2.376 | 0.001 | 2.212 | 0.002 | 2.067 | 0.001 |
|  | CF vs SL | 4.181 | 0.001 | 4.277 | 0.001 | 4.278 | 0.001 |
|  | CF vs CL | 4.283 | 0.001 | 4.188 | 0.001 | 4.338 | 0.001 |
|  | SF vs CMF | 1.815 | 0.002 | 1.675 | 0.004 | 1.639 | 0.008 |
|  | SF vs SL | 3.554 | 0.001 | 3.631 | 0.001 | 3.334 | 0.001 |
|  | SF vs CL | 3.128 | 0.001 | 3.156 | 0.001 | 3.025 | 0.001 |
|  | CMF vs SL | 2.780 | 0.001 | 2.672 | 0.001 | 2.546 | 0.001 |
|  | CMF vs CL | 2.700 | 0.001 | 2.635 | 0.001 | 2.722 | 0.001 |
|  | SL vs CL | 1.929 | 0.001 | 2.078 | 0.001 | 2.114 | 0.001 |
| Jaccard | CF vs SF | 1.183 | 0.010 | 1.145 | 0.039 | 1.137 | 0.044 |
|  | CF vs CMF | 1.551 | 0.001 | 1.422 | 0.001 | 1.356 | 0,003 |
|  | CF vs SL | 2.149 | 0.001 | 2.033 | 0.001 | 1.994 | 0.001 |
|  | CF vs CL | 2.443 | 0.001 | 2.308 | 0.001 | 2.280 | 0.001 |
|  | SF vs CMF | 1.315 | 0.001 | 1.243 | 0.003 | 1.177 | 0.02 |
|  | SF vs SL | 1.908 | 0.001 | 1.847 | 0.001 | 1.729 | 0.001 |
|  | SF vs CL | 1.966 | 0.001 | 1.903 | 0.001 | 1.799 | 0.001 |
|  | CMF vs SL | 1.587 | 0.001 | 1.500 | 0.001 | 1.430 | 0.001 |
|  | CMF vs CL | 1.677 | 0.001 | 1.598 | 0.001 | 1.591 | 0.001 |
|  | SL vs CL | 1.317 | 0.001 | 1.320 | 0.001 | 1.314 | 0.001 |
| Abundance weighted Unifrac (wei_unifrac) | CF vs SF | 0.838 | 0.571 | 0.915 | 0.483 | 1.069 | 0.327 |
|  | CF vs CMF | 5.954 | 0.001 | 5.357 | 0.001 | 3.737 | 0.004 |
|  | CF vs SL | 11.616 | 0.001 | 11.081 | 0.001 | 9.916 | 0.002 |
|  | CF vs CL | 8.812 | 0.001 | 8.213 | 0.001 | 8.022 | 0.002 |
|  | SF vs CMF | 5.734 | 0.001 | 5.833 | 0.001 | 4.542 | 0.003 |
|  | SF vs SL | 12.001 | 0.001 | 12.014 | 0.001 | 10.739 | 0.002 |
|  | SF vs CL | 8.348 | 0.001 | 8.515 | 0.001 | 8.529 | 0.002 |
|  | CMF vs SL | 5.357 | 0.001 | 4.634 | 0.001 | 4.429 | 0.002 |
|  | CMF vs CL | 4.780 | 0.001 | 4.101 | 0.001 | 4.154 | 0.002 |
|  | SL vs CL | 2.236 | 0.002 | 2.128 | 0.016 | 2.379 | 0.017 |

**Note:** Church forest (CF), state forest (SF), community managed forest (CMF), shrubland (SL), cropland (CL)

**Figures**

**Fig. S1** Mean monthly rainfall (mm), maximum (Tmax), and minimum (Tmin) temperatures (°C) from 2000 to 2022 (Adis Zemen meteorological station) of Tara Gedam Church Forest, northwest Ethiopia.

**Fig. S2** Schematic representation of heterogenous habitats in northwest Ethiopia

Note: CF represents the reference native forest dominated by indigenous large tree species and nutrient-rich soil. SF stands for naturally restored forest after the disturbances of Native forest by selective cutting of large trees and free grazing, dominated by indigenous trees and shrubs, sharing some species with CF. CMF refers to restored forest through both NR and enrichment plantation using exotic tree species after degradation through intensive illegal tree cuttings and free grazing, sharing some species with SF. SL represents degraded shrublands converted from the native forest due to deforestation and degradation, dominated by small shrubs and bushes, and characterized by poor soil and free grazing disturbance. CL refers to degraded cultivated lands gained after native forest deforestation with great share of area, covered with annual crops, and characterized by poor soil and tillage disturbances.

**Fig. S3** Rarefaction curves of soil prokaryotic ASVs (Amplicon Sequence Variants) across samples. Plateauing curves (a, Observed ASVs; b, Coverage; c, Shannon) indicate sufficient capture of most prokaryotic diversity. The vertical black dashed line indicates the rarefaction depth (1148 reads) used for downstream analyses.

**Fig. S4.** Alpha diversity of the soil prokaryotic community among habitats at rarefaction thresholds (2000 (a, b), and 3000 (c, d) denoised reads per sample). (a, c) Shannon’s and (b, d,) Faith’s phylogenetic diversity (PD) indices. Different lowercase letters indicate significant differences between habitats (P < 0.05) based on Dunn’s post hoc test following Kruskal–Wallis analysis.

**Fig. S5**. Alpha diversity metrics obtained from repeated rarefaction approach across habitat types (CF, SF, CMF, SL, and CL). Average Shannon diversity index (a) and phylogenetic diversity (PD) (b) for 50-time rarefaction at 1148 denoised reads per sample. Different lowercase letters above boxplots indicate significant differences among habitats (P<0.05) based on Dunn’s post hoc test following Kruskal–Wallis analysis.

**Fig. S6** (a) Unique and shared ASV counts across habitats. (b) Soil prokaryotic community structure across habitats based on PCoA using Jaccard distance. Ellipses represent group dispersion. PERMANOVA indicated significant differences among habitats (F = 1.703, p = 0.001), while PERMDISP showed no significant differences in dispersion (F = 1.7966, p = 0.126).

**Fig. S7.** Patterns of soil prokaryotic community structure among habitats across rarefaction thresholds of 2000 (a, b), and 3000 (c, d) denoised reads per sample. (a, c) Principal coordinate analysis (PCoA) based on Bray–Curtis and (b, d) weighted UniFrac distances. PERMANOVA For Bray–Curtis distances, F = 2.751022, P = 0.001; and F = 2.722245, P = 0.001 at 2000, and 3000 reads per sample, respectively. For weighted UniFrac distances, F = 6.163988, P = 0.001; and F = 5.639816, P = 0.001 at 2000, and 3000 reads per sample, respectively. Permutational analysis of multivariate dispersions (PERMDISP) indicated no significant differences in dispersion among habitats across rarefaction thresholds. PERMDISP for Bray–Curtis distances, F = 0.4149, P = 0.811; and F = 1.6536, P = 0.156 at 2000, and 3000 reads per sample, respectively. For weighted UniFrac distances, F = 0.9645, P = 0.436; and F = 0.4479, P = 0.795 at 2000, and 3000 reads per sample, respectively.

**Fig. S8** Differential abundance and environmental associations of prokaryotic taxa in church forest and adjacent habitats. (a) Linear discriminant analysis effect size (LEfSe) showing prokaryotic taxa (phylum to genus) significantly differing across habitats (LDA score > 4, P < 0.05). Colored nodes represent habitat specific-enriched taxa, with branch areas shaped by the most abundant taxa. (b) Spearman's correlations of the top 30 differential prokaryotic taxa per habitat with soil properties, stand characteristics, and indicator trees (* P< 0.05, ** P < 0.01, and *** P < 0.001). Church forest (CF), state forest (SF), community managed forest (CMF), shrubland (SL), cropland (CL)

**Fig. S9** Relative contributions of ecological processes to soil prokaryotic community assembly across habitats for all taxa. The Pie chart shows the proportional importance (%) of deterministic processes (homogeneous and variable selections) and stochastic processes (dispersal limitation, homogenizing dispersal, and ecological drift) in shaping prokaryotic communities.

**Fig. S10.** Robustness of βNTI/RCBray-based community assembly inference among habitats across rarefaction thresholds at 2000 (a, b), and 3000 (c, d) denoised reads per sample. Panels (a, c) show β-nearest taxon index (betaNTI) values, whereas panels (b, d) show Raup–Crick Bray–Curtis (RCBray) values. Different lowercase letters indicate significant differences among habitats (P < 0.05) based on Dunn’s post hoc test following Kruskal–Wallis analysis.

**Fig. S11.** Relative contributions of deterministic and stochastic assembly processes within each habitat at rarefaction depths of 2000 (a), and 3000 (b) denoised reads per sample. CF, church forest; SF, state forest; CMF, community-managed forest; SL, shrubland; CL, cropland.

**Fig. S12** The relative abundance (%) of predicted functions identified for all prokaryotic ASVs across habitats using FAPROTAX database

**Fig. S13** Relative abundance of dominant soil prokaryotic functions in church forest and adjacent habitats. Church forest (CF), state forest (SF), community managed forest (CMF), shrubland (SL), cropland (CL)

**Fig. S14** Spearman's correlation of dominant predicted functional groups with soil properties, stand characteristics, and indicator trees in church forest and adjacent habitats. Significant associations are indicated by P < 0.05 (*), P < 0.01 (**), and P < 0.001 (***). Abbreviations: *Teclea nobilis* (T_nob), *Albizia schimperiana* (A_schimp), *Cupressus lusitanica* (C_lus), *Maytenus arbutifolia* (M_arbut), stand density (density), basal area (BA), electrical conductivity (EC), total nitrogen (TN), bulk density (BD)
